# New treatments for advanced cancer: an approach to prioritization

**DOI:** 10.1054/bjoc.2000.1406

**Published:** 2000-10-26

**Authors:** J S J Ferguson, M Summerhayes, S Masters, S Schey, I E Smith

**Affiliations:** Lambeth, Southwark and Lewisham Health Authority, 1 Lower Marsh, London, SE1 7NT; Guy's King's and St Thomas' Joint Cancer Centre, London, SE1 7EH; The Royal Marsden Hospital NHS Trust, Downs Road, Sutton, SM2 5TP, UK

**Keywords:** cancer, treatment, prioritization

## Abstract

The allocation of funding for new anticancer treatments within the UK has not kept pace with demand. Clinicians find themselves restricted in the use of licensed drugs which they feel are in the best interests of individual patients. Against this, health authorities have a duty to ensure that scarce resources are used equitably to meet the needs of the local population as a whole. Differential levels of funding for new treatments across the country have led to concerns about rationing by postcode. This paper outlines an approach to the prioritization of new treatment for advanced cancer developed jointly by clinicians and health authorities in South London. The approach involves evidence reviews and consensus meetings. Existing and new treatments are rated on a four-point ‘relative effectiveness scale’, which takes account of the impact of the treatment on quality of life and on survival. The strength of evidence supporting each effectiveness rating is also classified. Health Authorities have used these ratings to determine overall funding levels, while leaving decisions on individual patients to the relevant Trusts. © 2000 Cancer Research Campaign


*British Journal of Cancer* (2000) **83**(10), 1268–1273
© 2000 Cancer Research Campaign
doi: 10.1054/ bjoc.2000.1406, available online at http://www.idealibrary.com on IDEAL®

### New treatments for advanced cancer: an approach to prioritization

**JSJ Ferguson[1], M Summerhayes[2], S Masters[1], S Schey[2] and IE Smith[3]**

[1]Lambeth, Southwark and Lewisham Health Authority, 1 Lower Marsh, London SE1 7NT; [2]Guy's, King's and St Thomas' Joint Cancer Centre, London SE1 7EH; [3]The Royal Marsden Hospital NHS Trust, Downs Road, Sutton, Surrey SM2 5TP, UK

**Summary** The allocation of funding for new anticancer treatments within the UK has not kept pace with demand. Clinicians find themselves restricted in the use of licensed drugs which they feel are in the best interests of individual patients. Against this, health authorities have a duty to ensure that scarce resources are used equitably to meet the needs of the local population as a whole. Differential levels of funding for new treatments across the country have led to concerns about rationing by postcode. This paper outlines an approach to the prioritization of new treatment for advanced cancer developed jointly by clinicians and health authorities in South London. The approach involves evidence reviews and consensus meetings. Existing and new treatments are rated on a four-point 'relative effectiveness scale', which takes account of the impact of the treatment on quality of life and on survival. The strength of evidence supporting each effectiveness rating is also classified. Health Authorities have used these ratings to determine overall funding levels, while leaving decisions on individual patients to the relevant Trusts. © 2000 Cancer Research Campaign

**Keywords:** cancer; treatment; prioritization

The use of chemotherapy for patients with cancer has increased markedly in the UK over the past few years (Richards and Parrott, 1996). In part this relates to the increased use of adjuvant chemotherapy following surgery for breast and colorectal cancer. Much of the increase, however, relates to the treatment of patients with advanced cancer. In the 1980s the use of chemotherapy was, in general, limited to patients with haematological malignancies, small cell lung cancer, breast cancer, ovarian cancer and rare cancers such as testicular teratoma and choriocarcinoma. More recently, chemotherapy has been given to a much larger number of patients with advanced stages of several common cancer types, including colorectal cancer, oesophagogastric cancer, non-small cell lung cancer and bladder cancer.

At least 12 new anticancer agents have been licensed in the UK in the past 3 years (Table 1) and more are expected to be licensed in coming months. Although in some instances these new treatments may be substituted for existing treatments, in other cases they are recommended as additional treatments. For example, there is likely to be an increase in the use of chemotherapy for non-small cell lung cancer (NSCLC) where none would have been given previously. Patients with advanced colorectal cancer whose disease has progressed following first-line chemotherapy may now be offered second-line treatment where none was previously available. The potential cost to the NHS of the new cytotoxic drugs alone could be considerable. The increase in total costs of care (including the costs of inpatient stays, day-case attendances, investigations, etc) if all these treatments become incorporated into clinical practice will be substantially greater.

*Received 23 September 1999*
*Revised 15 May 2000*
*Accepted 28 June 2000*

*Correspondence to:* JSJ Ferguson

Although expenditure on chemotherapy has risen considerably in the 1990s the allocation of additional resources has not kept pace with demand, especially for the new treatments. Clinicians find themselves restricted, either by Health Authorities or by provider Trusts, in the use of licensed treatments which they feel are in the best interests of an individual patient. Against this, Health Authorities have a duty to ensure that scarce resources are used equitably to meet the needs of the local population as a whole.

This paper outlines an approach to the prioritization of new treatment for advanced cancer developed jointly by clinicians and health authorities in South London. Our first aim was to achieve consensus regarding the relative effectiveness of a range of chemotherapy treatments given to patients with different types of cancer. Secondly, we wished to assess the strength of current evidence supporting the relative effectiveness rating for each treatment. Thirdly, we wished to present the information in a format which would enable health authorities to make rational decisions on the future allocation of resources.

In the future the National Institute for Clinical Excellence (NICE) may well have an active role in providing guidance on effectiveness and resource allocation in the UK. However, in the medium term they have to be addressed by clinicians and health authorities.

### METHODS

### Scope of project

The scope of the project was limited to treatment given for advanced cancer, as this encompasses the licensed indications of all of the new cytotoxic agents. Treatments given with curative intent (e.g. for some patients with acute leukaemias, lymphomas and teratoma) were excluded from this process as were adjuvant

**Table 1** New treatments

| Generic name | Trade name | Cost per cycle[a] £ | Tumour types for which the drug is licensed[b] |
|---|---|---|---|
| Docetaxel | Taxotere | 1560 | Breast cancer |
| Fludarabine | Fludara | 760 | B cell chronic lymphocytic leukaemia |
| Gemcitabine | Gemzar | 1030 | Non-small cell lung cancer<br>Adenocarcinoma of pancreas |
| Interferon α[c] | | | Various haematological malignancies |
| Irinotecan | Campto | 760 | Colorectal cancer |
| Liposomal Doxorubicin | Caelyx | 1070 | AIDS-related Kaposi sarcoma |
| Paclitaxel | Taxol | 1300 | Ovarian cancer<br>Breast cancer |
| Raltitrexed | Tomudex | 410 | Colorectal cancer |
| Rituximab | Mabthera | 1900 | Follicular lymphoma |
| Topotecan | Hycamtin | 1830 | Ovarian cancer |
| Vinorelbine | Navelbine | 175 | Non-small cell lung cancer<br>Breast cancer |
| Temozolamide | Temodal | 1380 | Glioblastoma multiforme |

[a] Costs per cycle have been calculated for a patient with a body surface area of 1.7 $m^2$ and are based on list prices including VAT. Cycles are typically repeated every 3–4 weeks, the total number of cycles depending on the patient's response to treatment and on toxicity; [b] Licensed indications for several of the new drugs are restricted to patients whose disease is resistant to standard treatments; [c] Interferon α – cost per cycle is not shown as the recommended dosages vary for different indications therapies (e.g. for breast and colorectal cancer). Endocrine treatments for patients with advanced cancer and supportive treatments given to patients receiving chemotherapy (e.g. antiemetics, antibiotics and colony-stimulating factors) have not been considered at this stage.

### Assessment of the relative effectiveness of treatments

The main objectives of giving anticancer treatments to patients with advanced cancer are to optimize quality of life (QoL) and, where possible, to prolong life. The overall effectiveness of a treatment cannot be determined by any single existing outcome measure, as none combines quantity and quality of life. The measures that are currently reported in clinical trials include:

- *Survival*: Does the treatment prolong median survival and if so by how long? The proportion of patients surviving for a specific interval after the initiation of treatment (e.g. 1 year, 2 years) is also sometimes reported.
- *Time to progression:* Does the treatment prolong the median time to disease progression and if so by how long?
- *Response rate*: What proportion of patients experience an objective response to treatment, measured in terms of a reduction in size of measurable lesions? This is principally a measure of drug activity against the cancer, but has been shown to correlate with improvement in QoL (Baum et al, 1980; Coates et al, 1987; Kaasa et al, 1988; Glimelius et al 1989; Ramirez et al 1998).
- *Quality of life*: How does the treatment impact on patients' QoL? The antitumour effect of the treatment may enhance QoL, while its toxicity may have adverse consequences for QoL. Several QoL measures have been specifically developed and validated for use in clinical research trials for patients with cancer (de Haes et al 1990; Aaronson et al 1993; Cella et al 1993).

Meta-analyses relating to the effectiveness of 'standard' treatments were used where available (NHS Executive, 1996; 1997; 1998; NSCLC Collaborative Group, 1995; Lilenbaum et al, 1998; Advanced Ovarian Cancer Trialists Group 1991; 1998). Reviews of the current published evidence related to the effectiveness of new treatments were undertaken independently by a senior Oncology Pharmacist (MS) and by consultants in Public Health Medicine. The information from these reviews was scrutinized by a panel of oncologists with extensive clinical and research expertise related to the relevant tumour types, to identify any important omissions or inaccuracies.

Relative effectiveness ratings were derived at consensus meetings, involving clinicians and health authority representatives, rather than being based solely on analyses of the published evidence. This approach was adopted for several reasons. First, we wished to combine the evidence relating to survival and quality of life into a single rating. Secondly, it was recognized that outcomes reported in the research literature apply to selected groups of patients included in clinical trials and may differ from those observed in routine clinical practice (Gregory et al, 1993). Thirdly, many early treatments for advanced cancer were not assessed in the context of randomized controlled trials (RCTs) with a control arm. The evidence is therefore suboptimal, but pragmatic decisions still need to be made to inform practice.

Participants in the consensus meetings were asked to consider where each treatment should be placed across a spectrum of effectiveness for palliative treatments, ranging from no benefit at one end to highly effective at the other. An example of a treatment with no benefit would be one with no impact on survival and where the toxicity, on average, offset any benefit in terms of relief of cancer-related symptoms. A four category scale (A – D) was used to categorize individual treatments, with the most effective treatments being assigned to category A and the least effective to category D.

Standard chemotherapy for small cell lung cancer (SCLC) was taken as an example of a highly effective palliative/life-prolonging treatment, which is widely recommended by clinicians (category A). The large majority of patients receiving treatment experience symptomatic benefit and improvements in QoL. Median survival is thought to be prolonged by about 9 months (based on comparisons with historical controls).

When new treatments were being compared with existing treatments a 'comparative effectiveness' rating was assigned (A – D). This represents the magnitude of the additional benefit of the new treatment over the established treatment:

A = Prolongation of median survival by > 9 months together with improvement in quality of life
B = Prolongation of median survival by 3–6 months with improvement in quality of life
C = Improvement in quality of life but little or no impact on median survival
D = Minimal impact on quality of life and no impact on median survival

### Strength of evidence

The strength of current evidence regarding the efficacy of new treatments has to be clearly differentiated from the magnitude of benefit/effectiveness. The following scale is used in this paper to denote strength of evidence.

α+ = Data from a meta-analysis or from at least two high-quality RCTs
α– = One high-quality RCT and supporting non-randomized (phase II) data
β = One poor-quality RCT and/or several phase II studies
γ = Single phase II study only
H = Survival evidence based on comparisons with historical controls.

### Standard treatments

For the purposes of this project, 'standard' treatments were defined as those regimens already used in clinical practice in South London which do not involve any of the recently licensed agents. The agents used in these regimens have been available for at least 10 years, although some of the combinations have only been introduced more recently. The term 'standard' should not be taken to mean that all patients with the relevant cancer should be recommended to receive the treatment. Rather, that these regimens have become accepted as appropriate for selected patients with the relevant cancer type.

### RESULTS

### Standard treatments

The list of treatments shown in Table 2 is not exhaustive, but relates to those cancer types for which most of the new treatments have been licensed.

Effectiveness ratings of 'A' were assigned to the first-line treatment of SCLC and follicular lymphoma. For breast cancer, first-line treatments were rated as 'B' and second-line treatment as 'C'. This reflects the lower response rates and shorter times to progression normally observed following second-line treatment. Standard first-line treatments for NSCLC were considered to be broadly similar in terms of effectiveness to standard second-line treatments for breast cancer.

The strength of evidence related to the individual effectiveness ratings for standard treatments largely reflects the era in which the respective treatments were introduced into clinical practice. Thus, for SCLC and breast cancer the evidence is based on extensive observational data related to response rates, time to progression and QoL parameters – but prolongation of life is not directly quantifiable owing to the lack of RCTs with a control arm. In contrast, sufficient studies of this type have been reported for patients with colorectal cancer and NSCLC to enable meta-analyses to be undertaken.

### New treatments

Table 3 shows the effectiveness and strength-of-evidence ratings for some of the newly licensed treatments. For most of the new

**Table 2** Effectiveness and strength of evidence for standard treatments

| Cancer type | Setting | Regimen(s) | Effectiveness rating | Strength-of-evidence rating |
|---|---|---|---|---|
| Small cell lung cancer | First-line | PE/CAV | A | β (H) |
| Non small cell lung cancer | First-line | MIC/MVP | C | α+ |
| Breast cancer | First-line | FAC/FEC | B | β |
| | Second-line | CMF/MV | C | β |
| Colorectal cancer | First-line | LVFU | B | α+ |
| Stomach cancer | First-line | ECF | B | α– |
| Ovarian cancer | First-line | CC/CAP/Carbo | B[a] | α+ |
| Follicular lymphoma | First-line | Chlorambucil | A | β |
| | Second-line | CVP/CHOP | B | β |

[a] Denotes effectiveness in comparison with non-platinum drug regimens; PE = cisplatin, etoposide; CAV = cyclophosphamide, doxorubicin, vincristine; MIC = mitomycin C, ifosfamide, cisplatin; MVP = methotrexate, vinblastine, cisplatin; FAC = fluorouracil, doxorubicin, cyclophosphamide; FEC = fluorouracil, epirubicin, cyclophosphamide; CMF = cyclophosphamide, methotrexate, fluorouracil; MV = mitomycin C, vinblastine; LVFU = leucovorin-primed fluorouracil; ECF = epirubicin, cisplatin, fluorouracil; CC = cisplatin, cyclophosphamide; CAP = cisplatin, doxorubicin, cyclophosphamide; Carbo = carboplatin CVP = cyclophosphamide, vinblastine, prednisolone; CHOP = cyclophosphamide, doxorubicin, vincristine, prednisolone

*© 2000 Cancer Research Campaign*

**Table 3** Assessment of new treatments

| Disease | Setting | New treatment | Comparator | Effectiveness of new treatment | Strength of evidence |
|---|---|---|---|---|---|
| Ovarian cancer | First-line | Paclitaxel and platinum | Various 'standard' | A[a] | α+ |
| | Second-line | Topotecan | Platinum | B[a] | α–/β |
| Follicular lymphoma | Third-line | Rituximab | – | A/B | β |
| Breast cancer | Second-line | Docetaxel | MV | B[a] | α– |
| Colorectal cancer | First-line | Raltitrexed | LVFU | C/D[a] | α+ |
| | Second-line | Irinotecan | Best supportive care | B | α– |
| Renal cancer | First-line | Interferon α | Medroxyprogesterone acetate | B[a] | α– |
| Kaposi sarcoma | Second-line | Liposomal doxorubicin | Doxorubicin | C[a] | α+ |
| Non-small cell lung cancer | First-line | Vinorelbine/cisplatin | Cisplatin | B[a] | α+ |
| | Second-line | Gemcitabine | Best supportive care | D | α+ |

[a] Denotes effectiveness in comparison with existing treatment; MV = mitomycin C, vinblastine; LVFU = leucovorin-primed fluorouracil treatments evidence of effectiveness is based on the results of randomized controlled trials where the comparator is an existing treatment. For these treatments the effectiveness ratings are based on the additional benefit observed compared with that of the existing treatment. The overall effectiveness of these new treatments (in comparison with best supportive care) can only be inferred. However, where existing treatments have been demonstrated to be more effective than best supportive care it might be argued that the overall effectiveness would be somewhat greater.

Only one new treatment (paclitaxel and platinum for first-line treatment of ovarian cancer) was rated as 'A' in comparison with standard treatments. The strength of evidence supporting this was 'α+'. Rituximab for relapsed follicular lymphoma was also given a high rating (A/B), but this was based on observational data (i.e. strength of evidence = β). Several other treatments were rated as 'B' or borderline B/C.

Liposomal doxorubicin was rated as 'C' for the treatment of Kaposi sarcoma, the lower toxicity associated with the new compound giving it an advantage over standard treatment with doxorubicin. Raltitrexed was rated C/D as there is no evidence of prolongation of life in comparison with leucovorin-primed fluorouracil, but the convenience of administration may be advantageous in some circumstances.

### DISCUSSION

The management of advanced cancer presents difficult decisions for patients and clinicians, quite apart from any considerations of financial cost. The treatments currently available frequently have only limited effectiveness and may have considerable toxicity. Predicting the levels of benefit and side-effects that individual patients will experience is extremely difficult, if not impossible. Clinicians have to be able to present the available evidence clearly, so that patients can weigh up from their own perspective the potential advantages and disadvantages of particular treatment options. Those responsible for clinical decision-making need to be mindful that patients facing a life-threatening illness may weigh the evidence differently from those in good health. In a study based on hypothetical scenarios patients with cancer were much more likely to opt for radical treatment with minimal chance of benefit than people who did not have cancer, including medical and nursing professionals (Slevin et al, 1990). In a recent study in the USA, patients who had received cisplatin-based chemotherapy for advanced NSCLC were asked whether they would accept chemotherapy given a range of hypothetical scenarios relating to toxicity and survival. Patients' willingness to accept chemotherapy varied widely, some accepting treatment for a survival benefit of only 1 week, others not accepting treatment even for 24 months prolongation of life. However, most reported that they would accept chemotherapy if it substantially reduced symptoms without prolonging life (Silvestri et al, 1998).

The evidence available to clinicians on the benefits and toxicities of individual treatments includes both the published literature from clinical trials and their own experience gained from treating previous patients. The approach adopted for this project represents an attempt to formalize this process by combining an objective assessment of the research evidence with the experience of a group of clinicians.

Decisions regarding the delivery of chemotherapy do not rest with clinicians and patients alone. Health authorities have to evaluate health care needs and competing claims for service developments across all health services, against a background of limited resources. In relation to advanced cancer health authorities have to decide whether additional resources will be made available both for extensions in the use of 'standard' chemotherapy treatments and for the introduction of new therapies. The potentially competing claims for resources for other palliative interventions and for specialist palliative care also have to be considered. At present, individual health authorities across the UK are undertaking separate reviews of the effectiveness of each of the newly licensed anticancer treatments and are making individual decisions on the allocation of resources. Variations in resource allocations have led to concerns regarding rationing by postcode.

The approach described in this paper involved a partnership between NHS Trusts and Health Authorities (Secretary of State for Health, 1997) and has, we believe, provided a rational basis for resource allocation. We have been able to achieve broad consensus between clinicians and commissioners in South London regarding the relative effectiveness of different chemotherapy treatments and the strength of evidence supporting these ratings. Those responsible for resource allocation have informed us that they find this approach helpful for their understanding both of the clinical issues and the large amount of data from individual studies. Additional funding has been made available based largely on the estimated costs of providing new treatments rated A or B for effectiveness and with an α+ or α– for strength of evidence. However, at an individual patient level decisions rest with provider units, thus avoiding blanket bans on specific treatments. Activity and

© 2000 Cancer Research Campaign 

outcomes related to the use of each of these new treatments are being audited. We believe that the methods and results reported in this paper should be transferable to other health care systems. However, the relative priority given to chemotherapy for advanced cancer may well differ between countries leading to the adoption of different thresholds for funding of new treatments.

Our approach has some similarities to that reported from Greater Manchester (Foy et al 1999), but also has some important differences. In particular we evaluated the magnitude of benefit and the strength of evidence separately, according to predefined scales, rather than simply categorizing treatments as being of proven clinical effectiveness over and above existing treatments. Unlike the Manchester Group we did not define a specific threshold for funding at our consensus meetings, thus avoiding pressure to move the threshold up or down.

We readily acknowledge that our work to date has limitations. Any method which attempts to combine effects on length of life and quality of life in a single measure involves value judgements regarding the relative importance of the two dimensions. In practice, most chemotherapy agents which have a significant impact on survival also have quality of life benefits, as both effects are mediated through a reduction in tumour burden. Some treatments may, however, have significant QoL benefits with only marginal effects on survival (e.g. through having lower toxicity than a previous treatment, but with both treatments having an equivalent impact on survival).

As yet we have only conducted limited work in relation to the assessment of costs. It is reasonably simple to estimate the likely additional costs per patient of the chemotherapy agents per se (see Table 1). It is also possible to estimate the likely number of patients within a given population who might match the licensed indications for the treatments. It is more difficult to estimate the proportion of patients who would wish to receive the treatment, particularly for treatments at the lower end of the effectiveness scale. The overall costs of treatment are likely to be substantially greater than the costs of the chemotherapy agents alone (Richards et al, 1993). Although the drug costs are highly visible the impact on hospital bed usage and on outpatient and day-case attendances (among other factors) may be of equal importance. Work which is currently in progress for the development of chemotherapy Healthcare Resource Groups (HRGs) will hopefully address this. The costs that would be incurred in caring for patients who do not receive a specific treatment also need to be considered.

At present the scope of this project has been limited to chemotherapy and biological therapies for advanced cancer. Extending the work to incorporate other new treatments for advanced cancer, such as new endocrine agents or new approaches in the delivery of radiotherapy should be quite simple. We believe that comparisons could also potentially be made with treatments for other advanced incurable illnesses, given to patients with limited life-expectancy. However, for treatments given with curative intent other approaches such as quality-adjusted life-years (QALYs) gained are more appropriate.

We hope that this paper will stimulate debate regarding the use of chemotherapy in patients with advanced cancer. Do oncologists, public health physicians and the pharmaceutical industry agree with our ratings of effectiveness? At what point should the strength of evidence be deemed adequate for decisions to be made regarding resource allocation? It might be argued that at least two RCTs are needed – the first being seen as hypothesis-generating and the second as providing confirmatory evidence. This standard may well be unachievable, especially for treatments given for rare cancers. The ethical and practical issues of recruiting patients with advanced cancer into a second RCT when the positive results of one RCT are known should also be considered.

### ACKNOWLEDGEMENTS

The authors acknowledge the support given to this project by the South East London Cancer Taskforce and thank the clinicians and health authority representatives who participated in the consensus meetings: Drs A Barnett, P Bevan, S Bowcock, B Bryant, D Cunningham, P Ellis, M Gore, P G Harper, R Ireland, N Lemic, D Miles, N Mir, Prof J Moxham, A Pagliuca, S Rassam, E Sawicka, J Spiby, R Stott and J Webb.

### REFERENCES

Aaronson NK, Ahmedzai S, Bergman B, Bullinger M, Cull A, Duez NJ, Fletchtner H, Filberti A, Fleishman SB and de Haes JC (1993) The European organisation for research and treatment of cancer QLQ-C30: A quality of life instrument for use in international trials in oncology. *J Natl Cancer Inst* **85**: 365–376

Advanced Ovarian Cancer Trialists Group (1991) Chemotherapy in advanced ovarian cancer: an overview of randomised clinical trials. *BMJ* **303**: 884–893

Advanced Ovarian Cancer Trialists Group (1998) Chemotherapy in advanced ovarian cancer: four systematic meta-analyses of individual patient data from 37 randomized trials. *Br J Cancer* **78**: 1479–1487

Baum M, Priestman T, West R and Jones E (1980) A comparison of subjective responses in a trial comparing endocrine with cytotoxic treatment in advanced carcinoma of the breast. In *Proceedings of the Second EORTC Breast Cancer Working Conference* pp 223–226. Pergamon Press: Oxford

Cella DF, Tulsky DS, Gray G, Sarafin B, Linn E, Bonoma A, Silberman M, Yellen SB, Winicour P and Brannon J (1993) The Functional Assessment of Cancer Therapy Scale: development and validation of the general measure. *J Clin Oncol* **11**: 570–579

Coates A, Gebski V, Bishop J, Jeal P, Woods R, Snyder R, Tattersall M, Byrne M, Harvey V, Gill G, Simpson J, Drummond R, Browne J, Van Cooten R and Forbes J (1987) Improving the quality of life during chemotherapy for advanced breast cancer: a comparison of intermittent and continuous treatment strategies. *N Eng J Med* **317**: 1490–1495

de Haes JCJM, van Kippenberg FCE and Neijt JP (1990) Measuring psychological and physical distress in cancer patients: structure and application of the Rotterdam Symptom Checklist. *Br J Cancer* **62**: 1034–1038

Department of Health (1996) Improving outcomes in breast cancer: The research evidence. Department of Health, London

Department of Health (1997) Improving outcomes in colorectal cancer: The research evidence. Department of Health, London

Department of Health (1998) Improving outcomes in lung cancer: The research evidence. Department of Health, London

Foy R, So J, Rous E and Scarffe JH (1999) Perspectives of commissioners and cancer specialists in prioritising new cancer drugs: impact of the evidence threshold. *BMJ* **318**: 456–459

Glimelius B, Hoffman K, Olafsdottir M, Pahlman L, Sjorden P and Wennberg A (1989) Quality of life during cytostatic therapy for advanced symptomatic colorectal carcinoma: a randomised comparison of two regimens. *Eur J Cancer Clin Oncol* **25**: 829–835

Gregory W, Smith P, Richards M, Twelves C, Knight R and Rubens R (1993) Chemotherapy of advanced breast cancer: outcome and prognostic factors. *Br J Cancer* **68**: 988–995

Kaasa S, Mastekaasa A and Naess S (1988) Quality of life of lung cancer patients in a randomised clinical trial evaluated by a psychosocial well-being questionnaire. *Acta Oncol* **27**: 335–342

Lilenbaum RC, Langenberg P and Dickersin K (1998) Single agent versus combination chemotherapy in patients with advanced non-small cell lung carcinoma. A meta analysis of response, toxicity and survival. *Cancer* **82**: 116–126

Non-small Cell Lung Cancer Collaborative Group (1995) Chemotherapy in non-small cell lung cancer: a meta-analysis using updated data on individual patients from 52 randomised clinical trials. *BMJ* **311**: 899–909

*© 2000 Cancer Research Campaign*

Ramirez AJ, Towlson KE, Leaning MS, Richards MA and Rubens RD (1998) Do patients with advanced breast cancer benefit from chemotherapy? *Br J Cancer* **78**: 1488–1494

Richards MA, Braysher S, Gregory WM and Rubens RD (1993) Advanced breast cancer: use of resources and cost implications. *Br J Cancer* **67**: 856–860

Richards MA and Parrott JC (1996) Tertiary cancer services in Britain: benchmarking study of activity and facilities at 12 specialist centres. *BMJ* **313**: 347–349

Secretary of State for Health (1997) *The new NHS: Modern Dependable*. HMSO: London

Silvestri G, Pritchard R and Welch HG (1998) Preferences for chemotherapy in patients with advanced non-small cell lung cancer: descriptive study based on scripted interviews. *BMJ* **317**: 771–775

Slevin ML, Stubbs L, Plant HJ, Wilson P, Gregory WM, Armes PJ and Downer SM (1990) Attitudes to chemotherapy: comparing views of patients with cancer with those of doctors, nurses and general public. *BMJ* **300**: 1458–1460
